# How U.S. Federal Artificial Intelligence (AI) policy is shaping agrifood systems: an integrative review

**DOI:** 10.3389/frai.2026.1881767

**Published:** 2026-07-22

**Authors:** Cole Baerlocher, Sarah McCord, Elizabeth Tabares, Arturo España, E. Alex Keasler, Rafael Landaverde

**Affiliations:** 1 U.S. House Ways and Means Committee, U.S. House of Representatives, Washington, DC, United States; 2 Department of Agricultural Leadership, Education and Communications, Texas A&M University, College Station, TX, United States

**Keywords:** agriculture, agrifood systems, artificial intelligence, integrative review, policy

## Abstract

Artificial intelligence (AI) is increasingly shaping how agrifood systems function in the United States, yet the role of federal policy in guiding its use, oversight, and broader consequences is still not well defined. This study explores how current U.S. federal AI policies support-or limit-the advancement of agrifood systems by synthesizing evidence from publicly available policy documents. Using a focused search strategy and qualitative content analysis, we reviewed nine federal policy documents released through September 2025 to identify key priorities and overlooked areas relevant to agriculture. Our analysis revealed six recurring themes: environment, precision agriculture, workforce development, governance, technological infrastructure, and partnership. The findings show that federal AI policy places considerable emphasis on building infrastructure, strengthening workforce capacity, and establishing governance frameworks. At the same time, less attention is given to environmental trade-offs, equitable access for small- and mid-scale producers, and the place specific conditions that shape agricultural practice. Notably, tensions emerge between policies that promote rapid expansion of AI infrastructure and those aimed at protecting environmental resources and strengthening climate resilience. Taken together, the results suggest that although agriculture is increasingly recognized within the national AI agenda, the lack of a coordinated, agriculture specific policy framework may lead to uneven adoption and unintended outcomes across the agrifood system. This study offers a synthesized policy foundation to support future research, inform decision making, and engage stakeholders in aligning AI innovation with more sustainable and equitable agrifood systems.

## 1 Introduction

Artificial Intelligence (AI) has been an emerging force in the transformation of new technology within the United States (U.S.) (Omotayo et al., 2025). Its influence has shaped not just the digital economy but also the agrifood system, creating an impact on production methods, climate variability, and national food security (Aijaz et al., 2025; Alexander et al., 2023; Da Silveira et al., 2026). As agricultural production grows its interest in automation, data systems, and new technologies, the need for federal policy is critical in identifying how AI is implemented, monitored, stored, and controlled (Alkoby and Hirschprung, 2026; Khanna et al., 2022). The implementation and development of strategic AI policy frameworks for the agriculture industry and agrifood system have implications such as guiding producers in enhancing operations, aiding predictive analyses, and optimizing resources (Aijaz et al., 2025). However, without holistic groundwork to guide automation in the agrifood system and the adoption of AI in agriculture, it leaves industry stakeholders, including farmers, researchers, and additional counterparts, with a lack of knowledge and confidence (Aijaz et al., 2025).

While many opportunities for increased productivity, sustainability, and efficiency exist through AI (Singh and Kaur, 2022), there are also important questions around data rights, environmental sustainability, access for small-scale producers, and the preparedness of rural areas to utilize these emerging technologies (Zheng et al., 2024). The U.S. federal government should understand that adopting AI policy will only succeed if every producer has access to these emerging tools, highlighting the need for strong frameworks that promote equity within the industry (U.S. Department of Agriculture, 2024). In recent years, the U.S. government has made AI research and AI strategic planning a key issue across all agencies. However, across multiple administrations, the agricultural industry has seen a variation in the attention it receives toward implementing AI frameworks. While current strategies acknowledge the potential AI has within the agrifood system, they rarely outline exact strategies for uplifting producers, improving rural technology deserts, or directing the creation of agriculturally focused AI tools (Omotayo et al., 2025). For example, in the Executive Order on Safe, Secure, and Trustworthy Development and Use of Artificial Intelligence, AI implementation is highlighted as a tool that should only take place in ways that protect Americans and promote the public interest (Executive Office of the President, 2023). This document broadly includes agriculture but does not explicitly mention AI innovation within the industry. These types of inconsistencies demonstrate a growing need to understand how federal AI policies engage with producer needs and how these decisions shape industry innovation (Romanishyn et al., 2025; Zheng et al., 2024).

Other insight comes from the limited federal funding and sponsorship opportunities for research on the advancement of intelligence automation (IA) in agriculture, which is outlined exclusively under the standard grant “Supporting Research at the Intersection of Agricultural Science, Big Data, Informatics, and Smart Communities” supported by U.S. National Science Foundation (NSF) and the U.S. Department of Agriculture‘s National Institute of Food and Agriculture (USDA/NIFA), with a strong emphasis in technological infrastructure, computing architecture, and analytics (National Science Foundation (NSF), U.S. Department of Agriculture National Institute of Food and Agriculture (USDA NIFA), 2019). Through a survey and revision of the U.S. policy decisions and initiatives considering the use and advancement of AI in agriculture, this study addresses the research question: (1) In what ways do emerging U.S. federal AI policy priorities enable or constrain agrifood systems advancement?

### 1.1 Review of literature

“Artificial Intelligence is social and cognitive phenomena that enable a machine to socially integrate with a society to perform competitive tasks requiring cognitive processes and communicate with other entities in society by exchanging messages with high information content and shorter representations” (Abbass, 2021).

The U.S. and the People’s Republic of China are the most capable players in the fierce competition for seizing the global leadership in AI (Meleouni and Giannou, 2025). Historically, the U.S. has loosely led in all areas of competence, but invincibility could change or remain unchanged depending on the strategic efforts (Meleouni and Giannou, 2025). According to the 2024 global AI vibrancy ranking developed by the interdisciplinary institute for the development of Human-Centered Artificial Intelligence at Stanford University, the remarkable superiority of the U.S. lies on five out of seven pillars: responsible AI (14.29 vs. 5.72), economy/investments (14.29 vs. 1.26), talent (7.21 vs. 4.89), policy and governance (10.86 vs. 0.65), and infrastructure (13.90 vs. 5.49) (HAI, n.d.). The other two criteria show a slight superiority of the U.S. on R&D (9.85 vs. 8.86) and of China on public opinion (7.27 vs. 8.19) (Fattorini et al., 2024; HAI, n.d.). Two of the pillars deserve a close look, given their importance at this stage. First, research and development (R&D) measured as AI scientific publications in SCOPUS was led by the U.S. until 2008, when it became first runner-up behind China, and second runner-up behind India in 2023 (OECD, n.d.). By 2025, the difference in publications was 22 percentage points lower than China and 4 percentage points lower than India (OECD, n.d.). For the second criterion, talent, the U.S. has been superseded by Singapore (7.98) and India (7.82) and ranked second in the share of women in AI publications behind China (HAI, n.d.; OECD, n.d.). An exploration of the topics addressed by national policies of IA leading countries, from an academic and AI practitioner’s perspective, shows that by 2018, R&D, talent acquisition, the future of employment skills, adoption of AI in strategic sectors, opening of datasets to encourage AI R&D, development of ethical standards, and addressing of complex social problems such as poverty and exclusion, were the national priorities among the 11 top nation’s leading AI development (Fattorini et al., 2024; Lauterbach, 2019).

A recent topical modelling analysis (Saheb and Saheb, 2023) of 30 national policies found the national priorities in the international arena fall in six categories in order of concerning: (1) educational, focused on digital skills and higher education, (2) technological, focused on high performance computing infrastructure, deep learning technologies, autonomous systems and transport, and cyber security, (3) governmental focused on open public data and data sharing, and algorithmic decision-making. The last three categories have less visibility, attention, or restricted views: (4) ethical and legal, with references to legal aspects of data protection, ethical principles, and socio-economic risks and public trust, (5) new players, such as AI startups and the companies within the AI system, along with the automation of labor markets, and finally (6) social good such as healthcare and environmental and climate-friendly living that lay in the sustainability of the agriculture, energy, and transportation sectors (Saheb and Saheb, 2023).

AI policies in the U.S. are among the most abundant in the leading nations (Fattorini et al., 2024; Meleouni and Giannou, 2025). The OECD inventory accounts for a total of 84 U.S. records in all categories and sectors, with a national strategy, 11 national AI governance bodies or mechanisms, 26 regulations, guidelines, and standards, and 46 AI policy initiatives, programs, and projects (OECD, n.d.). Still, the debate on the approach is open and divided between the supporters of “permissionless innovation” who see regulation as a threat to it and advocate for soft law alternatives (Thierer et al., 2017), and those who are concerned about risks and demand strong innovative governance and regulation to mitigate the negative aspects of AI advancement (Siau and Wang, 2018).

Some sectors in the U.S. have achieved a certain level of consensus to formulate their policies aimed at facilitating IA development in areas like public governance, innovation, digital economy, education, defense, and healthcare (OECD, n.d.). However, agriculture is a lagging sector with a high possibility of remaining behind since there is no overarching policy or legislative framework to guide and boost the future of an AI-enhanced agrifood system (Da Silveira et al., 2026). This lag is reflected not only on the scarce internal policy visibility within the U.S. but also in the international arena as the estimates of venture capital (VC) financial investment in AI firms worldwide show (OECD, 2021). Based on private-source data from Preqin, agriculture venture deals represented less than 1% of the venture investment deals in all industries worldwide between 2012 and 2020, with only 170 deals out of 20,549 (OECD, 2021). Lauterbach (2019) suggests that the trends from venture capital deals lean toward the application of AI in precision agriculture through agricultural robots, in-field monitoring using drones and deep-learning-based computer vision, crop and soil health analytics, satellite imagery analysis, predictive analytics, and technologies to reduce damage.

Considering technological advancements, previous literature, and the current lack of structured policy to support the integration of AI-driven agrifood systems, we completed an integrative literature review to understand the emergent AI-agricultural policy priorities established by the U.S. government.

## 2 Materials and methods

This integrative review followed the procedures proposed by Gusto and Diaz (2020) and was informed by Torraco’s (2005) integrative literature review method to identify and examine public policy documents produced by the federal government focused on AI in agrifood systems. Torraco (2005) defines an integrative review as a form of research in which scholars act as the research instrument by reviewing, critiquing, and synthesizing relevant literature on a particular topic, and later integrating the evidence into a new analytical perspective or framework (Torraco, 2016).

Even though Torraco’s recommendations have been widely used in integrative review studies, his approach recognizes that contextual elements and the needs of a particular study will influence how researchers organize their review of the literature (Torraco, 2016). Despite this flexibility, several researchers emphasize that conventions for conducting an integrative review must be clearly described, including how documents were selected, from the search strategy to the reporting of findings (Toronto and Remington, 2020; Torraco, 2016).

Our methodological decisions were heavily influenced by researchers who have successfully implemented detailed guidelines to leverage literature resources and generate new analytical insights (Dhollande et al., 2021; Hopia et al., 2016). In response to the purpose of this study, and considering the way public policy documents are created and disseminated through governmental websites and publicly owned repositories, we implemented a deliberate and targeted search strategy, rather than the broader approach typically recommended for academic literature reviews, since these documents are not systematically indexed or published under scientific database standards.

With respect to artificial intelligence applied to agrifood systems, the need for a deliberate and targeted search becomes even more pronounced, as this is an emerging area of interest whose developments and guidelines appear first in institutional documents, technical reports, strategic roadmaps, and regulatory frameworks—long before they become peer-reviewed literature. For this reason, we conducted a structured document search designed to capture relevant federal policy documents and institutional reports. The search strategy was developed iteratively and included combinations of keywords such as: *“Artificial Intelligence policy,” “federal program in artificial intelligence,” “USDA artificial intelligence policy/report,” “artificial intelligence legislation,” “White House artificial intelligence initiative,” and “Congress bill for artificial intelligence.”* Because policy documents are not consistently indexed in traditional academic databases, we conducted targeted searches across official U.S. government repositories and agency websites. These included, but were not limited to: congress.gov, whitehouse.gov, and usda.gov.

While it is not possible to conduct an exhaustive search of public policy documents through academic databases, our efforts focused on strategic actors and institutional repositories commonly responsible for generating the most up-to-date and relevant evidence for policymaking. This strategy not only acknowledges the dispersed and heterogeneous nature of these sources but also ensures that the documents captured the institutional narratives, priorities, and tensions that shape the advancement of AI in agrifood systems.

Relevant resources were organized using a spreadsheet, recording the authoring organization, areas of implication, and the implications identified. By utilizing this organizational scheme, the analysis and presentation of new evidence became easier and more effective (Gusto and Diaz, 2020). The search concluded in September 2025, and documents were assessed for themes using a qualitative content analysis approach introduced since the 1980’s (Krippendorff, 2019). Content analysis provided researchers with a systematic procedure for assessing the included documentation for themes (Erlingsson and Brysiewicz, 2017).

Upon reviewing and organizing the documents, the data were systematically sorted into thematic categories by two coders following stablished content analysis procedures (Neuendorf, 2017). An initial coding framework was developed based on the research objectives and refined inductively as new patterns emerged from data. Both coders independently applied the coding scheme to the dataset and then met regularly to compare coding decision and resolve discrepancies by reaching consensus (Creswell, 2007). Any disagreements were resolved through discussion and iterative refinement of code definitions to ensure consistency and clarity.

Themes were subsequently reorganized and refined through an iterative analytical process that included continuous comparison across coded segments and ongoing interpretation of the data. To enhance credibility and transparency, the coders engaged in member checking and maintained analytic memos documenting coding decisions and theme development. This process continued until thematic saturation was achieved and full agreement on the final set of themes was reached (Neuendorf, 2017).

Documents were included if they met the following criteria: they were produced by a U.S. federal agency or official government body, directly addressed artificial intelligence, represented formal policy instruments, and were available in full text. Exclusion criteria included documents that were not directly relevant to artificial intelligence, secondary commentary (e.g., opinion pieces or summaries), documents lacking sufficient detail for analysis, and duplicate or superseded versions of the same document. The initial screening was based on titles and summary descriptions to assess relevance. This was followed by a full-text review to confirm eligibility and alignment with the study’s analytical framework. During the full-text review stage, additional exclusions were made when documents did not sufficiently address the study focus or failed to meet the previously defined inclusion criteria.

A total of nine full-text documents were included in the final examination section [S. 507 Bill - The Promoting Precision Agriculture Act of 2025, The Safe, Secure, and Trustworthy Development and Use of Artificial Intelligence (Executive Office of the President, 2023)], USDA Fiscal Year 2025–2026 AI Strategy, Accelerating Federal Permitting of Data Center Infrastructure (Executive Office of the President, 2025), USDA Compliance Plan for MB Memoranda M-24-10 - September 2024, Promoting The Export of the American AI Technology Stack (The White House, 2025b), Advancing United States Leadership in Artificial Intelligence Infrastructure (Executive Office of the President, 2025), Winning the Race: America’s AI Action Plan (The White House, 2025c), Promoting the Use of Trustworthy Artificial Intelligence in the Federal Government (Executive Office of the President, 2023). Each document was published by the federal government to understand how the U.S. governs the incorporation of agricultural AI policy into its broader AI agenda and implications for the agrifood system. Despite the current hype and public interest in AI, the number of available public policy documents remains limited. As AI innovations continue to be integrated into agrifood systems, and as the sociocultural, economic, and political contexts surrounding AI in agriculture continue to evolve, it will be important to revisit this study and expand the corpus of policy documents included in future analyses.

## 3 Results

**Figure 1:**
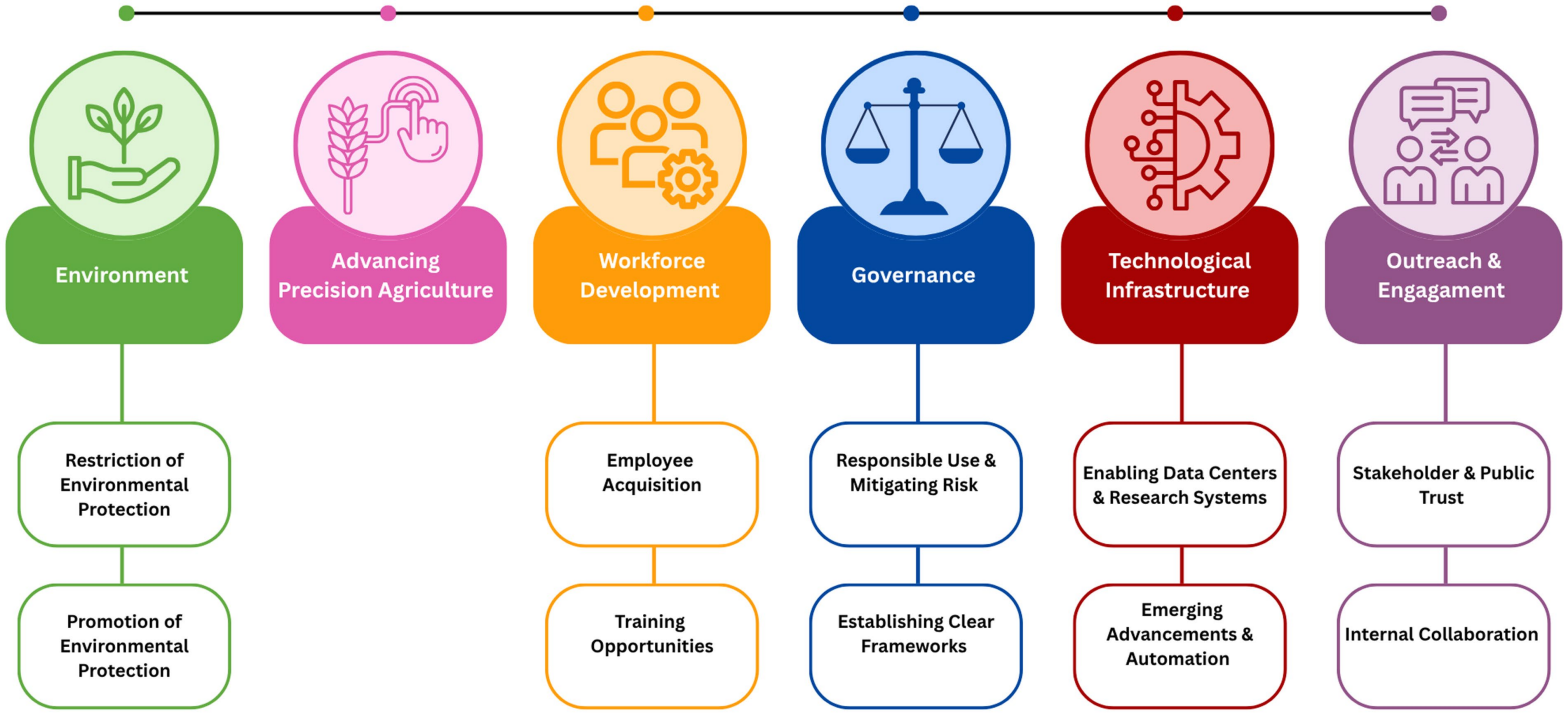
Six themes and respective sub-themes identified from the analysis.

This study aimed to understand key themes and implications of AI use for the advancement of agrifood systems. Concluding the analysis, six main themes emerged including: *Environment*, *Advancing Precision Agriculture*, *Workforce Development*, *Governance*, *Technological Infrastructure*, and *Partnership*. Each of these themes identifies how current policy emphasizes AI adoption and what gaps exist within the legislation. The concentration of these findings creates an understanding of how AI policies can influence agricultural stakeholders and what opportunities exist for improving national oversight of AI within the agricultural industry. A total of 141 statements were collected for the extraction of themes, and one statement was excluded due to a lack of relevance to the research question and objective of the study upon the refinement of themes. Table 1 allocates each identified theme, defined subthemes, and the total count of statements for each primary theme and subtheme. Figure 1 presents the emergent themes through a data visualization showing the distribution of themes and coordinating sub-themes.

**Table 1:** Emergent AI themes & subthemes in U.S. federal policy documentation.

Theme	Sub-themes	Total sub-theme count
Environment (*n* = 23)	Restriction of environmental protection	10
	Promotion of environmental protection	13
Advancing precision agriculture (*n* = 11)	n/a	11
Workforce development (*n* = 27)	Employee acquisition	12
	Training opportunities	15
Governance (*n* = 30)	Responsible use & mitigating risk	13
	Establishing clear frameworks	17
Technological infrastructure (*n* = 31)	Enabling data centers & research systems	18
	Emerging advancements & automation	13
Outreach & engagement (*n* = 18)	Stakeholder & public trust	10
	Internal collaboration	8

This table describes the major themes discovered upon analysis of AI policy documents.

Each theme is described in depth in the following sections and expanded accordingly.

### 3.1 Environment

Policy surrounding environmental ramifications drove the establishment of the Environment theme, which was a prominent topic across several study documents, making up (*n* = 23) extracted statements. This theme showcases the role that environmental regulations and sustainability goals play in the proactive adoption of AI from the USDA. Within this theme, two key sub-themes emerged: Restriction of Environmental Protection and Promotion of Environmental Protection. These two opposing sub-themes showcase the U.S. policymaking process, especially considering the contentious nature that environmental issues play within the agricultural industry. As political agendas shift depending on the nature of the democratic electoral process, policies, objectives, and goals within documentation change too, as suggested by the contrasting sub-themes in the Environment theme.

#### 3.1.1 Restriction of environmental protection

The Restriction of Environmental Protection sub-theme appeared the most often in documents that highlighted the benefits of creating a streamlined process for regulations related to technological development. The current administration, under President Trump, beginning January 2025, has released legislation that reduces environmental protections and allows federal lands to be available for data center construction by federal agencies with significant land portfolios (The White House, 2025a). Although these regulations are meant to increase the global competitiveness of the U.S. in AI development, they consequently have a negative impact on environmental safety and ecosystem sustainability, which remains both a necessary component for agricultural areas to continue production and develop habitable resources. By reducing federal oversight, the negative impact of land use, water availability, and damage to ecological systems could be factors that affect the long-term sustainability of the agricultural industry.

Statements in this sub-theme (*n* = 10), notably enhanced the development process of AI infrastructure with specificity to the environmental utility associated with the construction of data centers, etc. Likewise, this theme specifically draws attention to information published in the Clean Water Act Section 404, the National Environmental Policy Act (NEPA), the Endangered Species Act (ESA), the Environmental Response, Compensation, and Liability Act, and more. In this context, the restriction of environmental protection indicates loosening the regulations of the previous policies established by the pre-coursing administration so as to increase the feasibility and ease of development for the adoption of AI practices throughout U.S. agriculture (Alexander et al., 2023).

#### 3.1.2 Promotion of environmental protection

Conversely, the Promotion of Environmental Protection sub-theme contrasts with the Restriction of Environmental Protection sub-theme by showcasing how AI has the potential to strengthen climate-smart production practices, improve conservation, and further develop environmental monitoring systems. Former policies established between 2021 and 2025, under the Biden administration, go as far as promoting the procurement of clean energy generation resources as byproducts of AI infrastructure (Executive Office of the President, 2023). These initiatives align with the governmental goal of building up the agricultural industry’s ability for climate resilience, soil health, and regenerative land management practices. Through this perspective, AI can be viewed as a tool that is fully capable of improving the environment and advancing production systems. Statements in this theme (*n* = 13) prioritize advancing AI with respect to maintaining a conscious effort towards climate resilience. Clean energy, the promotion of minimal adverse effects on the environment, and the evolution of AI with consideration to ecosystem variability and resistance were advocated for in such documentation represented by this theme.

However, the turmoil between the rapid advancement of technology and the need for environmentally conscious practices is evident in the entire theme of Environment, and consequently, the two sub-themes indicating the restriction and promotion of AI in agrifood systems. On one hand, the federal government aims to maintain global dominance within the sector through accelerated infrastructure development. Conversely, the agricultural industry is deeply driven by production and ecosystem conservation, requiring stable environmental protections to maintain productivity and concern for the land. The contradiction in environmental policy may result in confusion or conflict for producers, technological advisors, and federal agencies when implementing AI initiatives and advancing the agrifood system through AI infrastructure.

For agricultural producers, strong environmental oversight has a direct impact on not just one’s ability to incorporate AI technology into their operations, but also on how implementation occurs. The future of environmental policy in the context of AI technological advancement both contributes to and hinders producers’ access to water, quality soil, and climate stability. Thus, environmental policy remains a foundational issue in determining the direction of AI integration within the agrifood system and governance of this topic in the U.S., paired with differing political agendas among various stakeholders and presidential agendas.

### 3.2 Advancing precision agriculture

The Advancing Precision Agriculture theme totaled (*n* = 11) statements which centered around directly enhancing agricultural practices through AI adoption. Statements extracted from the documentation in this theme highlight priorities driven by the Promoting Precision Agriculture Act of, 2025, including improvements in minimizing livestock and crop input losses. The U.S. Senate developed legislation that strives to enhance the participation of producers in precision agriculture in the United States and to establish voluntary consensus standards within the industry (S. 507, 119th Cong., 2025). Precision agriculture is defined in USDA policy (S. 507, 119th Cong., 2025) and others (Taylor, 2023) as a management strategy that employs technological automation using satellite navigation tools and other digital monitoring systems to increase the performance of agricultural production. Precision agriculture is emphasized for its improvement to efficiency, risk management, and enhanced capabilities in climate-smart practices.

Defined by the U.S. Department of Agriculture (2024), AI-based decision systems are mechanisms that aid producers in modifying their operations to prepare for variable weather patterns, reduce waste, and improve overall productivity. Whether to improve efficiency in seed, water, time, fuel, emissions, etc., achieving precision agriculture is a goal set by the federal government and its frameworks around food security, sustainability, and environmental resilience, as suggested by the previous theme as well. While federal policies mainly refer to AI innovation from an all-encompassing perspective, precise verbiage and defining characteristics of precision agriculture remain underdeveloped, including a lack of directness within these policy documents reviewed for this study. Despite this, the frameworks which appear in government agendas provide insights into the role federal policies have in breathing life into AI within production agriculture. AI tools such as automated technologies, yield analytics, pest detection, and robotic planting, watering, and harvesting have become beneficial within the precision agriculture movement. These tools rely on integrated data with machine learning models that work to optimize resources and lessen production costs.

Despite the imminent benefits AI has for implementing precision agriculture techniques, there are still challenges that threaten the incorporation of AI technology and its emergence. Recent strategies do not directly account for the broad scope of farm sizes, differing cropping systems, or regional conditions that impact what precision tools are able to be adopted by producers. Without strong regulations that focus on the dissemination of these technologies to different groups, producers may run into major obstacles when aligning AI technology with the reality of many operations. The farmers who run into the most difficulties are small-scale producers in areas with limited resources. These kinds of farmers will have to overcome barriers such as cost, access to infrastructure, and education on the implementation of these tools.

### 3.3 Workforce development

Another major theme across the reviewed documentation was the governmental desire to increase the capacity of the American workforce through training and improved employee hiring, recruitment, and acquisition. The theme, Workforce Development, consisted of (*n* = 27) total statements divided between two major themes, Employee Acquisition (*n* = 12) and Training Opportunities (*n* = 15). Mentions within this theme highlighted improvements in how developing human resources and capital can aid in reaching better outcomes for AI adoption within agriculture. Current federal policies refer to the need to grow the AI workforce pipeline through competitive hirings, science, technology, engineering, and mathematics (STEM) education resources, recruiting experts through incentives, and increasing the AI literacy and skill development of American agriculturists (The White House, 2025a).

#### 3.3.1 Employee acquisition

The focus of the theme of Workforce Development and the subtheme of Employee Acquisition was specifically centered around hiring, recruiting, retaining, and acquiring employees capable of leveraging AI resources in the agricultural sector. This theme revealed how the federal government has grown its work on building new initiatives that help to develop talent within every agency. AI policies acknowledge a national emphasis on including AI among talent growth, highlighting a commitment to building human capacity while incorporating new technologies in both agricultural work and life. With the recruitment of new employees, the digital mindset of the workforce increases, while simultaneously encouraging students and early career professionals to engage in AI adoption and implementation. For example, the USDA Digital Service plans to utilize the Pathway’s intern program and other programs such as the Virtual Student Federal Service (VSFS) and Coding It Forward to contribute to building an AI-competent set of interns while simultaneously creating a “talent acquisition bench,” to direct-hire employees when positions become available at the federal level (U.S. Department of Agriculture, 2023). By connecting the talent pipeline with the agricultural industry, the federal government helps to set the industry on track to ensure the agrifood system operates in a more cost-effective and efficient manner. AI policy emphasizes the government’s desire to acquire new employees capable of meeting the demands of the digital workforce, while also maintaining the human element of the agricultural industry, so as not to fully automate the sector, but optimize the productivity of the agricultural sector with the addition of AI.

#### 3.3.2 Training opportunities

Beyond strictly acquiring new state of the art employees with AI skillsets, the USDA and other governing agencies suggest that training and skill development opportunities will also be key to advancing AI in agriculture with existing employees. The reviewed literature suggests USDA and associated entities have a strong commitment to providing unique opportunities to current employees to learn through new training modules, educational resources, and cross-agency collaboration for professional development. The USDA’s Agricultural Research Service’s (ARS’s) scientific computing program, SCINet, is working to develop and deliver a variety of AI-related training resources for ARS researchers on data science and the fundamentals of scientific computing (U.S. Department of Agriculture, 2023). AI policy emphasizes a commitment for leveraging data science analytics, university partnership, and opportunistic digital advancements to enhance the utility of AI for agricultural employees. While recruiting employees with already managed AI skill development provides an opportunity for modern diffusion of AI, the agricultural sector and its agenda setters also understand the importance of enhancing and furthering the human capital of internal employees.

Workforce development strategies aimed at improving the technological savvy skillset of agricultural employees narrows the competitiveness of the U.S. within the AI sector comparative to other nations while tying such an agenda to the federal government simultaneously. Building an AI component workforce will be established both internally and externally through increased learning opportunities for current employees, and a moment of acquiring skilled AI professionals to join the ever-advancing agricultural sector. Thus, policy highlighted here underscores the importance of improving training resources for federal employees.

Further, within the agricultural industry, the workforce shortage has proven to be a major challenge in developing existing skills to incorporate AI. This threat reduces the industry’s ability to improve rural areas, provide competitive wages, and expand the ability to have highly trained AI professionals. Likewise, producers encounter higher costs and barriers to employing and accessing new technology, particularly in the support of operation and management techniques; if adequate resources are not available to educate producers on the operation of new technologies, maximized utility from AI may be lost within the industry. Thus, with appropriate recruitment and workforce development for the adoption and application of AI techniques, the agricultural industry will better be able to support environmental, precision agriculture, and other advanced initiatives imposed by society and government alike.

### 3.4 Governance

Next, we identified AI Governance as a major theme among the reviewed AI policy documentation. AI Governance incorporates nuances from previous themes combining the prioritization of workforce development to create strategies promoting responsible and ethical AI readiness among USDA and agricultural industry employees. However, Governance was distinguished by two subthemes: Responsible Use & Mitigating Risk (*n* = 13) and Establishing Clear Frameworks (*n* = 17), encompassing 30 statements with relevance to Governance across included policy documents. As emphasized in America’s AI Action Plan, “AI must be developed and deployed in a manner that advances innovation while ensuring safety, security, and equity” (The White House, 2025a), reinforcing the need for more intentional integration of agriculture within national AI governance.

#### 3.4.1 Responsible use & mitigating risk

The sub-theme Responsible Use & Mitigating Risk builds on previous themes discussed within Workforce Development and the coordinating sub-theme Training Opportunities. We build on previous themes here, suggesting that to ensure proper adoption of AI technologies within the agricultural workforce, training must be provided, but through training opportunities, outlined parameters must be instilled to guide the responsible and ethical initiation of AI use throughout agriculture and consequently, the agrifood system. Policy recommends that AI adoption should be beneficial and equitable to support American employees, so they have a welcome chair at every table (Executive Office of the President, 2023). Documentation suggested that the governance of AI should be coordinated through responsible measures to create inclusive adoption among Americans ranging from developers to small business owners, educators, labor unions, entrepreneurs, and every worker across the U.S. (Executive Office of the President, 2023). Policy calls for a “fair, open, and competitive ecosystem and marketplace” to drive AI innovation (Executive Office of the President, 2023). Initiatives here ensure that AI may be accepted in a reasonable manner to maintain ethical adoption, data readiness, compliance, security, and risk management across AI use and distribution platforms.

Further, policy describes that embracing AI in the agricultural workforce “should not be deployed in ways that undermine rights, worsen job quality, encourage undue worker surveillance, lessen market competition, introduce new health and safety risks, or cause harmful labor-force disruptions,” (Executive Office of the President, 2023). Thus, responsible use also brings mitigating risks and provides assurance for safe and secure AI operations within the industry. Though not a specified sub-theme we extracted, reviewed policy documents also built on the responsible use of AI by suggesting that cybersecurity efforts specifically should be ensured upon the dissemination and adoption of AI within the agricultural industry. Executive Order 14100 (Executive Office of the President, 2023) wrote that soundly promoting and implementing AI, “requires addressing AI systems’ most pressing security risks - including with respect to biotechnology, cybersecurity, critical infrastructure, and other national security dangers - while navigating AI’s opacity and complexity,” (para. 5). Thus, ensuring responsible use of AI throughout the sector would leverage digital resources and enhance the training opportunities and professional development governed by the federal government and related USDA agencies.

#### 3.4.2 Establishing clear frameworks

Additionally, the sub-theme Establishing Clear Frameworks supports previous themes as well, suggesting that responsible use of AI driven by appropriate workforce development techniques will be best performed under the guidance of clear adoption standards and frameworks. Principles of this theme are grounded in the government’s desire to empower the American workforce through clear, flexible standards outlined in a transparent governance framework. The USDA suggests, “AI governance standards and policies will help USDA’s workforce effectively utilize AI by clarifying expectations for AI development and deployment while enabling exploration” (U.S. Department of Agriculture, 2023). Beyond outlining minimal expectations, policy also details the need to have clearly defined techniques for the design, development, and acquisition of digital tools over the evolution of technological systems and new information technologies as they are introduced or span an AI product’s lifecycle. Further, federal policy reviewed for this analysis underscores the importance of maintaining relevancy through continuous refinement of AI frameworks and an effort to make AI accessible, traceable, and practicable to enable others in recreating effective AI practices.

Both the federal government’s drive for effective training as an essential tool for responsible AI usage and maximization of employee opportunities through oversight in governed frameworks contributes to developing a standardized understanding of the AI implementation process, leading to elevated workplace efficiency for the U.S. These goals strive to improve the federal workforce to manage emerging technological needs. The former administration (under President Biden from 2021 to 2025) affirmed that “promoting responsible innovation, competition, and collaboration will allow the United States to lead in AI and unlock technology’s potential to solve some of society’s most difficult challenges” (Executive Office of the President, 2023). Continuing to develop responsible and ethical AI frameworks with clarity will prove to be critical to increase efficiency within AI governance and maximizing the potential for incorporation by the agricultural industry.

### 3.5 Technological infrastructure

With 31 mentions, the Technological Infrastructure theme had the highest number of statements found. This theme represents two sub-themes, including Enabling Data Centers & Research Systems (*n* = 18) and Emerging Advancements & Automation (*n* = 13). Technological infrastructure as an emergent theme was a prevalent theme and demonstrated how improved infrastructure can influence the incorporation of AI at the national level by leveraging advancements in automation.

#### 3.5.1 Enabling data centers and research systems

Through the theme emergent process, a strong emphasis on the physical construction of data centers and building computing capabilities that support AI development was exceedingly present. Initially, we allocated this theme to be defined only by the fabrication of increasing physical AI infrastructure; however, upon continued analysis of statements, we defined this theme to simultaneously include infrastructure related to data and research capabilities as well. With 18 statements, this sub-theme, Enabling Data Centers and Research Systems, was the most represented, documenting what planned or idealistic groundwork should enable the future of AI to be at the forefront of agrifood systems transformation. The federal government, specifically the USDA, has acknowledged the need for investing in data management practices to support AI readiness, unlock experimentation, and provide confidence in AI outcomes (U.S. Department of Agriculture, 2024).

Building on constructed infrastructure, the USDA aims to utilize resources to promote AI-driven research and development toward high-impact, mission-focused usage solutions. With efficient and effective spaces equipped with proper technology and resources, researchers and federal agencies can be best positioned to inform policy, research, and other areas, providing actionable insights into technical global solutions. Reviewed policy suggests infrastructure aims to be increased through land management and resource allocation for the construction of data centers and energy equipment (transmission lines, generators, dispatchable baseload sources, nonrenewable energy pipelines, transformers, and more). Additionally, the USDA plans to expand and develop AI infrastructure, including the USDA AI Lab, to enable mission-focused and high-impact AI use within the agency (U.S. Department of Agriculture, 2024). By integrating agricultural policy priorities into the development of AI infrastructure, the industry has a strong ability to adopt new innovations and leverage AI towards the service, prosperity, and security of Americans (The White House, 2025a; The White House Office of Science and Technology Policy, 2018).

#### 3.5.2 Emerging Advancements & Automation

An additional sub-theme in the Infrastructure theme was Emerging Advancements & Automation (*n* = 13). This sub-theme highlights the need for leveraging infrastructure and pairing automation and technological advancements with AI to enhance resources such as broadband and secure data systems, both essential elements for precision agriculture and advancing the agricultural industry. Similarly, policies focused on advancing the agricultural industry through innovation strive to advance technology that contributes to mobile networks, next-generation Wi-Fi services, or future networks using advanced technologies (S. 507, 119th Cong., 2025). In addition, we highlight the USDA’s goal of leveraging data analytics and automated tools, including geospatial mapping, advanced software analytics, pilot testbeds for AI systems, drones, wireless communications technologies, robotics, automated vehicle and fleet systems, and more machine-to-machine operations to expand the infrastructure and digital capacities of employees and the agricultural workforce.

Leveraging such technologies also comes with the need to invest monetarily to fund the expansive toolset, which the government plans to utilize. Researchers have emphasized how important federal investments are to the development of AI, both financially and technologically, especially considering the agrifood system as a major beneficiary of said investments. In short, Technological Infrastructure as a theme, including sub-themes Enabling Data Centers & Research Systems and Emerging Advancements & Automation, underscores the value and importance of investing in automations and combined infrastructure to advance agricultural development within the agrifood system to meet the needs of the growing population and globalization of the U.S. society.

### 3.6 Outreach & Engagement

Finally, the last emergent theme identified was Partnership with emergent sub-themes in Internal Collaboration (*n* = 7) and Stakeholder & Public Trust (*n* = 11). Both sub-themes reflect the importance of collaboration, coordination, and engagement while adopting AI and emerging technologies to enhance the agrifood system from sources incorporating internal federal partnership and external collaboration with the public and additional stakeholders.

#### 3.6.1 Internal collaborations

Cross-agency partnership was noted across many of the documents reviewed for our analysis, suggesting that to initiate effective AI adoption, federal organizations must assume the role of team players, too, hence the sub-theme Internal Collaborations. Multiple federal agencies have already come to partner together to create a unified approach to incorporating AI technologies. The USDA plans to collaborate with several federal agencies, such as the National Institute of Standards and Technology (NIST) and the Department of Energy, to share progress and best practices on AI topics (U.S. Department of Agriculture, 2024). Additional collaborative efforts are to occur between committees, including the Committee on Commerce, Science and Transportation, the Committee on Agriculture, Nutrition, and Forestry of the Senate, and the Committee on Science, Space, and Technology, and the Committee on Agriculture of the House of Representatives. Here, a multidisciplinary partnership between federal agencies occurs while combining federal voices from both legislative bodies of the U.S. Congress. In agreement with government policy reviewed, we suggest that upon mutually beneficial federal agency collaboration, the more suitable AI will be for public interest and mission-specific goals, which global stakeholders have (Romanishyn et al., 2025).

#### 3.6.2 Stakeholder and public trust

Similarly, the U.S. Senate introduced legislation that would promote coordination with relevant public and trusted private sector stakeholders to progress innovation within agricultural technology (S. 507, 119th Cong., 2025). Thus, the sub-theme Stakeholder & Public Trust highlights policy objectives to ensure that the adoption of digital technologies, such as AI, is met with trustworthiness and acceptance from the public. Trust in this context is defined by respect and confidence-building efforts when adopting digital technologies, whether in partnership with government entities, private sector stakeholders, voluntary consensus organizations, or foreign adversaries. Specifically, the reviewed policy aims to support partner countries in adopting American AI systems to aid in their developmental process as foreign partners and developing innovative regulatory frameworks and conducive infrastructure environments. Similarly, policy states that local, state, tribal, and territorial public beneficiary administrators implement the use of algorithmic systems and automated technologies for benefits systems. Thus, stakeholder engagement towards AI adoption is a multifaceted system requiring initiation at levels including local, state, and federal resources.

Further, documents suggest a keen focus on ensuring that relationships between stakeholders are utilized to solicit feedback, provide input, and employ AI in ways that enhance the working capital and employability of the agricultural workplace and the future workforce. The USDA recognizes that strong partnerships must be established to not only adopt AI technologies but also improve digital models with certainty and validity. USDA policies suggest their agency is exploring the idea of incentivizing opportunities for public error hunting to test and refine AI products with a customer-forward perspective. Refinement in this way allows not only public engagement to gain consumer confidence, but also maintains the element of partnership, which can often be lost in governmental objectives and agenda priorities.

Additionally, public partnership also focused on stakeholder engagement by extending the opportunity to collaborate with universities and private institutions in workforce development opportunities. These partnerships encourage the acceleration of innovation and help with the transfer of knowledge between the government and the public, including the transfer of knowledge, abilities, skills, and more. Strengthening these partnerships improves AI access for producers, agricultural extension employees, and agricultural researchers alike.

Finally, the Partnership theme showcases how the federal government is working to collaborate with stakeholders and set the pathway for success and trustworthiness within AI implementation across every sector of the agricultural industry, including collaboration from various partners across federal agencies, the private and public sectors, and more. This theme demonstrates the multidisciplinary nature of facilitating AI adoption through governmental policy and is representative of the heterogeneity that constitutes the agrifood system and transformation needed to maintain each portion of the sector, from food production to science and technology (Select Committee on Artificial Intelligence of the National Science and Technology Council, 2023).

## 4 Discussion

This study, using an integrative literature review and content analysis, examined nine United States federal government AI policy documents to understand the current state of incorporating artificial intelligence into federal policies and the implications these policies have on agrifood systems in the United States. The policy themes mentioned most frequently are those including technological infrastructure, workforce development, and governance, highlighting the shift in government priorities towards promotion of AI within the economy and the perceived value that the adoption of AI has for stakeholders (Alexander et al., 2023; Romanishyn et al., 2025). If these themes continue to guide federal action, they have the potential to create new job opportunities for precision agriculture educators, technology developers, and engineers, many of whom may be directly or indirectly supported by the federal government.

Policies addressing themes such as the environment, partnership, and advancing precision agriculture are acknowledged less frequently in published federal policies yet remain significant points to improve environmental outcomes and accelerate innovation within rural communities across the United States. The themes of environment and advanced precision agriculture are both themes which suggest that AI technology in agriculture can be a tool to increase yield and decrease waste of water, pesticides, and fertilizer. Less chemical use and subsequently less chemical runoff, along with less water waste from the aid of precision agriculture, could have a positive impact on the environment, landowners, and farm workers (Getahun et al., 2024). However, the subtheme of the restriction of environmental protection illustrates the trade-offs and highlights the use of public lands for data centers and the large amount of water that running AI requires to cool expansive data centers. Consumable amounts of clean water usage could have a long-term negative impact on agricultural systems’ access to clean water and the natural ecosystem services that may be removed by destroying public lands (Ingrao et al., 2023).

Contradictions regarding environmental regulations for artificial intelligence (AI) represent significant challenges for agricultural producers and implementing agencies. These policy tensions create uncertainty in decision-making, as stakeholders must navigate shifting priorities related to environmental compliance, conservation incentives, and production goals. For instance, producers may receive conflicting signals regarding investments in conservation practices versus productivity-enhancing strategies, while agencies must reconcile competing mandates across program implementation. This fragmentation can lead to inconsistent program delivery, reduced policy coherence, and challenges in long-term planning.

Political cycles play an active role in shaping these contradictions, often resulting in substantial shifts in environmental priorities within federal policy. For example, in October 2024, the Biden (2024) Administration’s National Security Memorandum on Artificial Intelligence (NSM-25), while primarily focused on national security, incorporated environmental safeguards. In contrast, in June 2026, the Trump Administration signed the National Security Presidential Memorandum on Artificial Intelligence in the National Security Enterprise, which rescinded and replaced NSM-25. This pattern suggests that AI-related environmental policy is highly sensitive to political turnover, raising concerns about policy durability and stability. As a result, stakeholders may perceive federal environmental policy as episodic rather than continuous, which can undermine long-term planning and sustained adoption of environmentally aligned practices.

To cope with these contradictions, producers can adopt flexible or “no-regret” management practices, such as ecosystem health improvements, soil management, and water-efficiency technologies. These actions align with conservation goals while also supporting productivity, thereby reducing vulnerability to policy shifts (Peake and Robb, 2021). In addition, producers can benefit from Extension services and technical assistance providers, which play a critical bridging role by translating evolving policy signals into clear, actionable guidance and helping producers interpret trade-offs in real time (Lázaro et al., 2025). At the institutional level, federal and state agencies can improve inter-agency coordination and harmonize guidance to ensure that programs deliver consistent signals to producers, even when broader policy priorities shift (Olomola, 2015). Finally, long-term program design (e.g., multi-year conservation incentives or contracts) can help buffer stakeholders from short-term political changes, increasing stability and encouraging the sustained adoption of environmental practices (Lázaro et al., 2025).

Despite the relevance of these strategies, their accessibility is not uniform across producers, underscoring important equity considerations for small- and mid-scale operations. Even though, policies incentivize innovation, they often do not sufficiently account for the structural differences and capacity of small-and -mid scale producers to adopt and integrate these technologies (Hazell et al., 2010). A clear example of these discrepancies is on precision agriculture, in which technologies require high upfront investment and advanced technical skills for the long-term implementation, which more commonly affect small scale farming operations (John et al., 2023). Similarly, the equity issues emerged across rural areas where connectivity infrastructure limits the implementation of innovations (John et al., 2023).

Similarly, the lack of capital and technical knowledge have created a historical trend of disadvantage that continue to affect small-scale producers’ access and participation in federally supported programs aimed at promoting agriculture development (John et al., 2023; Manono, 2025). Although these disparities reflect a broader historical trend in the United States, the documents analyzed do not consistently propose explicit mechanisms to support equitable access. While general federal policy documents rarely detail cost-sharing provisions or technical assistance models, those authored by the USDA do refer to such supports; however, they typically lack specificity regarding implementation. Moreover, these mechanisms are not systematically embedded across policy areas nor sufficiently tailored to address the unique needs of small-scale producers.

To address these gaps, future policy should prioritize the development of targeted support mechanisms for small- and mid-scale producers, including scaled cost-sharing programs, tailored technical assistance, and tiered participation requirements that reflect differences in operational capacity. Embedding these elements directly into policy design would improve accessibility and reduce structural barriers to participation.

### 4.1 Limitations

As stated in The Executive Order on Safe, Secure, and Trustworthy Development and Use of Artificial Intelligence, AI should be a tool used in ways that protect Americans and promote the public interest (Executive Office of the President, 2023). However, the policies examined in our integrative literature review and qualitative content analysis did not largely cover themes of data risks or ethical concerns, and broader ethical implications, especially if they relate to producers, rural communities, and small-scale operations. This suggests that the current federal AI policy governance is still in an early stage when it comes to the agrifood system.

These findings show that federal AI policy is beginning to recognize agriculture as part of the national agenda. As AI capabilities evolve and political priorities shift, revisiting and expanding this analysis will be essential for understanding where the U.S. agricultural AI policy stands. Limitations of the study include that only nine United States policy documents were examined, which were collected up to September 2025, and as expected the current AI policy landscape is rapidly evolving to cope with the AI’s development speed. The use of artificial intelligence in agrifood systems is a relatively new topic being covered in governmental policies; therefore, policy is not specific and rather contextual and derived from other areas of national interest.

### 4.2 Recommendations

Policies are constantly being published, and current policies that have been published after September 2025 may have conflicting goals or themes, like conflicting themes found throughout this analysis. As AI capabilities evolve and the political administrations change, the language used may shift dramatically as well. This language change may represent a shift in priorities, allocation of funds, and focus for the country. It is recommended that future research should periodically revisit this topic to examine how themes on AI-agriculture policy in the United States develop over time.

Tracking the priorities within the United States government throughout the years, pertaining to AI, is not only important for US stakeholders, but it could also be important for competing countries. In America’s AI Action Plan, “Winning the AI race will usher in a new golden age of human flourishing, economic competitiveness, and national security for the American people” (The White House, 2025a, pg. 1). We recommend future studies compare the policies in the United States to those of other countries that follow the lead with agricultural technology. While the policies covered in this research topic are limited to those published by the United States government and cannot be generalized, the information published could benefit the development of AI policies for agricultural systems in the context of other countries and panels.

AI policies federally published in the United States rarely focus on the operating size of agrifood systems to which artificial intelligence may be implemented. These policies are usually written and directed at large agricultural operations, with no mention of small-holder operations. It is recommended that researchers keep track of verbiage used in these policies to understand the possibilities for a technological gap between large- and small-scale farmers; this gap may be increased by the implementation of innovations that may not be understood or affordable to small-scale farmers.

Highly relevant topics for AI in agrifood systems remain under addressed in current United States policy frameworks. For example, data rights and privacy have emerged as critical constraints for agricultural stakeholders seeking to adopt AI technologies within their operations; however, these issues are not explicitly addressed in the policies analyzed. Similarly, cybersecurity represents a growing concern among both users and non-users of AI, further complicating adoption decisions. Together, these gaps highlight important areas where existing AI policies fall short and underscore the need for more comprehensive and actionable policy frameworks that ensure the long-term sustainability of innovation in agrifood systems.

Future research should expand the scope of analysis to include a larger and more up-to-date set of policy documents to better capture the rapidly evolving nature of AI governance in agrifood systems. There is also a need to incorporate validation with policymakers, Extension professionals, and agricultural stakeholders to strengthen the practical relevance of findings. Comparative studies across countries could provide insights into alternative policy approaches and best practices. Moreover, future work should more explicitly examine issues related to data rights, privacy, and cybersecurity, which remain under addressed in current policies. Finally, research should prioritize equity-focused analyses to better understand how AI-related policies can more effectively support small- and mid-scale producers.

## 5 Conclusion

As AI technology advances and develops, so does the U.S. federal government’s goal of implementing innovative technologies and incorporating artificial intelligence into the agricultural industry and complex agrifood system. Our study indicates that the U.S. government is committed to integrating AI across the country within the agricultural industry, despite the limited number of preestablished policies and currently available frameworks. Given the heightened integration of AI in the agrifood system, this study provides an overview of the current landscape and an outlook for continued multi-actor adoption of AI in agriculture.
